# Global and Regional Diagnostic Results of Progress Toward Cervical Cancer Elimination, According to the WHO Strategy: A Systematic Literature Review with Narrative Synthesis

**DOI:** 10.3390/diagnostics16081224

**Published:** 2026-04-20

**Authors:** Dan Cristian Luca, Ciprian Cirimbei, Sinziana Octavia Ionescu, Vlad Rotaru, Dan Nicolae Straja, Mihnea Alecu, Elena Chitoran, Daniela Cristina Stefan, Laurentiu Simion

**Affiliations:** 1 Medicine School, “Carol Davila” University of Medicine and Pharmacy, 050474 Bucharest, Romania; dan-cristian.luca@drd.umfcd.ro (D.C.L.); ciprian.cirimbei@umfcd.ro (C.C.); sinziana.ionescu@umfcd.ro (S.O.I.); vlad.rotaru@drd.umfcd.ro (V.R.); dannicolaestraja@gmail.com (D.N.S.); mihnea.alecu@umfcd.ro (M.A.); elena.chitoran@drd.umfcd.ro (E.C.); laurentiu.simion@umfcd.ro (L.S.); 2 General Surgery and Surgical Oncology Department I, Bucharest Institute of Oncology “Prof. Dr. Al. Trestioreanu”, 022328 Bucharest, Romania

**Keywords:** WHO strategy, cervical cancer, global strategy, HPV screening, cervical cancer screening

## Abstract

**Background/Objectives**: In 2020, the World Health Organization (WHO) launched a strategy to eliminate cervical cancer by ensuring high vaccination coverage, effective screening, and access to treatment. This review assesses how this strategy has been implemented in different world regions, what results have been achieved, and what disparities and challenges have been faced. **Methods**: A systematic search was performed in PubMed, Web of Science, GLOBOCAN, IARC, ASCO, and ESMO for publications issued between November 2020 and December 2024. A total of 721 records were reviewed and assessed, resulting in the inclusion of 47 studies in the final analysis. **Results**: Considerable regional variations were identified between the regions analyzed. Countries with organized vaccination programs, structured screening systems, and stronger treatment infrastructure generally reported more favorable outcomes. In contrast, several settings in Eastern Europe, parts of Asia, and sub-Saharan Africa remained below the WHO targets, with lower vaccination uptake, limited screening participation, and a persistently high cervical cancer burden. Progress was further constrained by inequalities in access to healthcare, differences in program organization, and limited comparability of available national data. **Conclusions**: Although many countries demonstrate that eliminating cervical cancer is achievable, global alignment with WHO targets remains uneven. Strengthening health infrastructure, improving vaccination uptake, and ensuring equitable access to screening and treatment are essential to turning this strategy into a realistic global outcome.

## 1. Introduction

**Figure 1:**
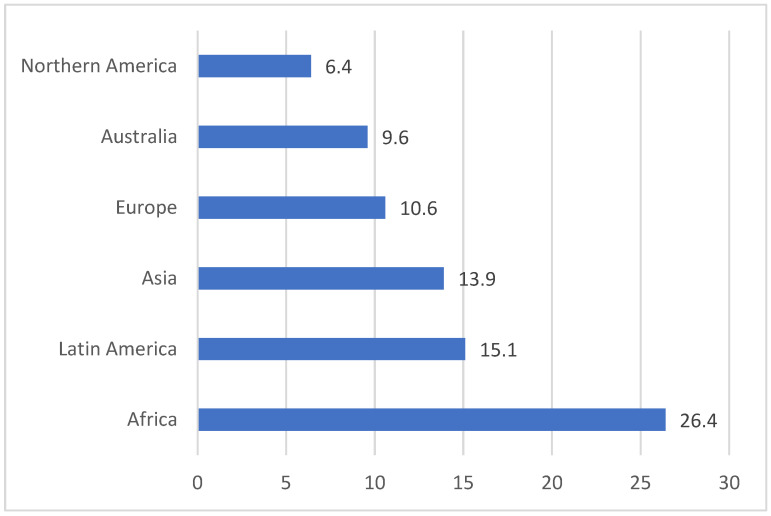
Cervical cancer incidence by region. Age-Standardized Rate (World) per 100,000, incidence, in 2022 (source: GLOBOCAN 2022 [2]).

Cervical cancer is a major global health problem and is ranked fourth among cancers in women. According to GLOBOCAN statistics from 2022, at least 600,000 new cases of cervical cancer are diagnosed annually, and 340,000 deaths associated with this disease are identified [1]. The incidence of this disease is in constant dynamics, with significantly higher frequencies in regions where incomes are low or middle, and access to screening and vaccination programs remains limited (Figure 1).

**Figure 2:**
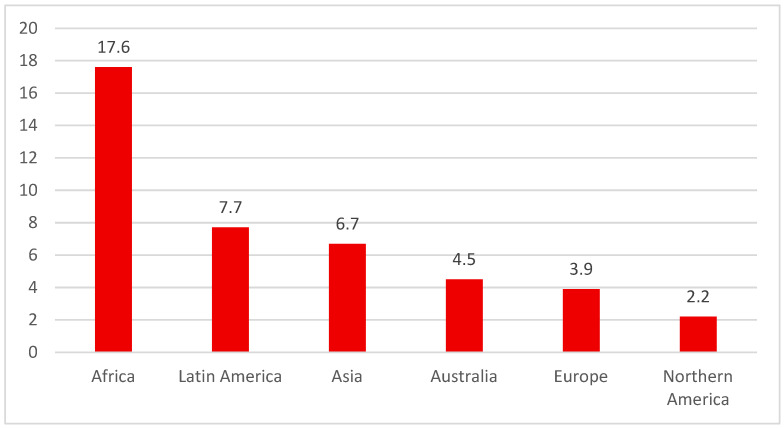
Cervical cancer mortality by region. Age-Standardized Rate (World) per 100,000, mortality, in 2022 (source: GLOBOCAN 2022 [2]).

Regarding the incidence and mortality of cervical cancer, the International Agency for Research on Cancer (IARC) confirms the presence of geographical differences between them, emphasizing the importance of prevention and equitable access to quality medical services (Figure 2).

**Figure 3:**
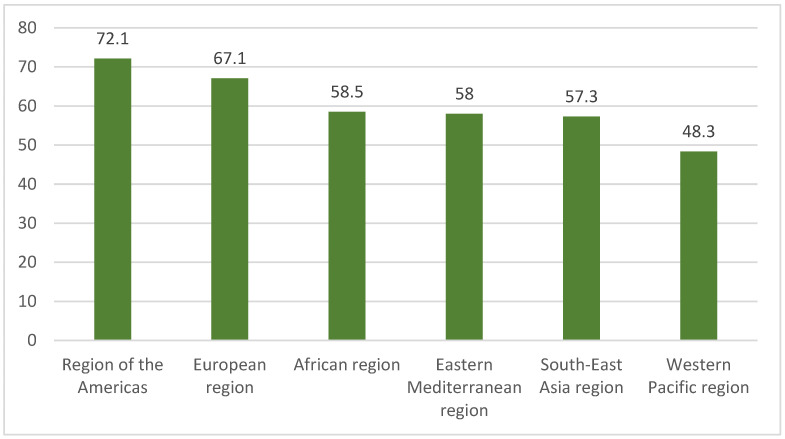
Estimated HPV vaccination coverage by region in the target female population in 2023 (%).

Screening is a key element in reducing the incidence and mortality of cervical cancer and has substantially reduced disease burden in countries with organized screening programs [3,4]. However, Pap testing and HPV testing are not equivalent as primary screening methods, and HPV testing is currently recommended by the WHO as the preferred primary screening approach [5,6,7]. Another key element in the fight against this public health problem is HPV vaccination, which remains a vital component of cervical cancer prevention [8,9]. In many regions, the main challenge is not vaccine ineffectiveness, but low vaccine uptake and limited implementation of vaccination programs [9]. With adequate uptake and sufficient follow-up, HPV vaccination can reduce HPV infection, high-grade cervical lesions, and ultimately cervical cancer in women vaccinated before HPV exposure [8,9] (Figure 3).

In 2020, the World Health Organization (WHO) launched a global strategy to eliminate cervical cancer as a worldwide health problem, “Global Strategy to Accelerate the Elimination of Cervical Cancer as a Public Health Problem”. This strategy is based on three essential pillars: vaccination against HPV infection of 90% of girls aged 9 to 14; access to screening for 70% of women aged 35 to 45; and treatment of 90% of women diagnosed with precancerous lesions or invasive cervical cancer [10,11].

The WHO strategy recommends a comprehensive approach to the problem presented, integrating good collaboration between the private sector, regional, and international governmental organizations. Measures aimed at improving access to HPV vaccines, assimilating financial funds for the development of the medical industry and awareness campaigns, improving screening programs, and training medical personnel [12].

Therefore, considering all these key elements, it is possible to eliminate cervical cancer as a significant public health problem in the near future [13].

## 2. Materials and Methods

This systematic literature review followed the PRISMA 2020 guidelines and aimed to evaluate progress in implementing the WHO strategy for eliminating cervical cancer.

To obtain a comprehensive and balanced understanding of how the WHO strategy is implemented across different regions worldwide, we reviewed international databases, including PubMed, Web of Science, GLOBOCAN, and IARC, as well as the ASCO and ESMO databases, to identify relevant epidemiological data, official reports, policy documents, and guidelines related to cervical cancer prevention and implementation of the WHO strategy.

To identify studies and reports related to cervical cancer and alignment with the WHO strategy, Boolean search terms were used to combine search terms, and the syntax was adapted to each database.

The PubMed database search was performed using the Boolean strategy with queries like: (“cervical cancer” [Title/Abstract] OR “cervical carcinoma” [Title/Abstract]) AND (“WHO” [Title/Abstract] OR “World Health Organization” [Title/Abstract] OR “global strategy” [Title/Abstract] OR “elimination strategy” [Title/Abstract]) AND (“HPV” [Title/Abstract] OR “HPV vaccination” [Title/Abstract] OR “cervical screening” [Title/Abstract] OR “HPV testing” [Title/Abstract]), applying filters to limit results to publications issued between November 2020 and December 2024.

The search in the Web of Science database was performed using the Advanced Search function, combining the following terms: (“cervical cancer” OR “cervical carcinoma”) AND (“WHO” OR “World Health Organization” OR “global strategy” OR “elimination strategy”) AND (“HPV” OR “HPV vaccination” OR “cervical screening” OR “HPV testing”), with filters applied to identify publications issued between November 2020 and December 2024.

Because GLOBOCAN, IARC, ASCO, and ESMO do not support a structured search format like PubMed and Web of Science, multiple specific manual searches were performed, using combinations of the following terms: “cervical cancer”, “WHO strategy”, “HPV vaccination”, “cervical cancer screening”, “HPV screening”.

After completing the database and manual searches, the retrieved records were analyzed according to predefined inclusion and exclusion criteria. Articles and reports were considered eligible if they explicitly addressed the implementation of the WHO strategy to eliminate cervical cancer; included information on HPV vaccination, cervical cancer screening, and treatment, as well as national outcomes or progress indicators; and were published between November 2020 and December 2024, being available in full text and in English. Studies were excluded if they did not explicitly refer to the WHO strategy, were not related to the elimination of cervical cancer, or if the full text was unavailable or access was restricted.

Because the aim of this review was to assess global and regional progress toward implementation of the WHO strategy, the eligibility criteria included original studies, registry-based reports, official documents, guidelines, and selected review articles when they provided relevant epidemiological or implementation-related data.

Following the selection criteria, in the first stage, a total of 721 articles were identified and analyzed based on their titles, abstracts, and the information presented. Finally, for this work, 47 articles were selected and grouped by regions such as Asia, Africa, Europe, Australia, South America, and North America, for clearer regional comparison and interpretation.

Data extraction was performed by one reviewer, using a form written in Word format. To reduce potential errors, all extracted information was subsequently checked for accuracy by another senior team member. There were no disagreements during study selection. However, in case of potential disagreements, it was decided to resolve them through discussion and, if necessary, by consulting a third independent reviewer. No further clarification was required from the authors of the included studies, as all relevant information was published in the published reports.

The obtained information was synthesized and analyzed, focusing on progress toward the WHO strategy objectives, vaccination results, screening program, and cervical cancer treatment.

The completed PRISMA 2020 checklist is provided in the Supplementary Materials Table S1.

## 3. Results

### 3.1. Study Selection

**Figure 4:**
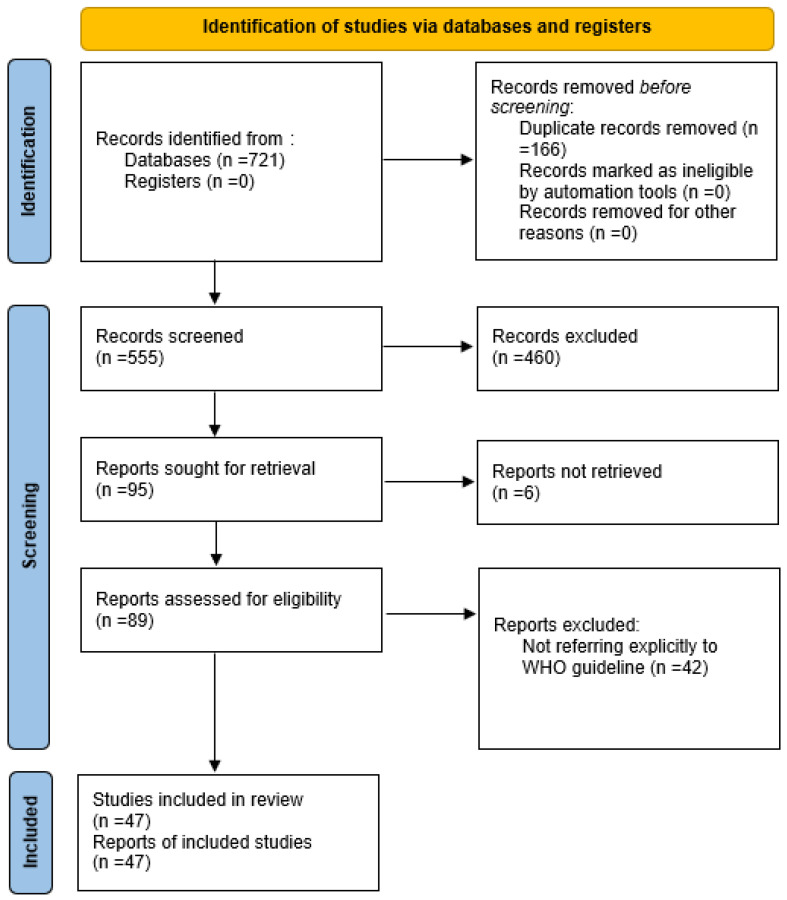
PRISMA 2020 flow diagram.

The study selection process is illustrated in Figure 4 (PRISMA 2020 flow diagram), and Table 1 presents the characteristics of the publications included in the review. Initially, 721 records were identified via database searches. After removing 166 duplicates, 555 records were screened based on their titles and abstracts, leading to 460 exclusions for being irrelevant to the WHO strategy. The remaining 95 reports were retrieved for full-text review, although 6 could not be accessed in their entirety due to paywall restrictions or unavailable or inaccessible full sources; they were thus excluded from the analysis. In the end, 47 studies satisfied the inclusion criteria and were included in this review.

### 3.2. HPV Vaccination

According to the available data, the implementation of the WHO strategy still requires major efforts in the development and expansion of HPV vaccination programs, contributing to marked disparities between high-income and low-income countries [14,15,16]. In settings with low vaccination coverage, the main barriers include population hesitancy, limited healthcare infrastructure, financial constraints, and the lack of effective public health campaigns [14,15,16]. At the same time, countries with high vaccination coverage have reported substantial reductions in cervical cancer incidence, particularly when coverage approached 90% among girls aged 9 to 14 years [14,15,16,17]. Latin America has also shown that integrating vaccination efforts with information, training, and community engagement activities can improve coverage and contribute to declining cervical cancer rates [18,19,20,21].

Favorable results were also recorded at the European level in countries such as Italy, which highlighted a coverage of 71% in girls and 55% in boys by 2022 through the development and improvement of vaccination programs, and the United Kingdom, which achieved HPV vaccination rates of 85% among 13-year-old girls. In contrast, Romania or Poland face barriers in vaccination coverage (below 30%), which is directly associated with increased incidence [22,23,24,25,26,27].

In Japan, HPV vaccination coverage was high in some pre-suspension birth cohorts but declined sharply after the suspension of proactive governmental recommendation in 2013 and remains substantially lower in many subsequent cohorts [28]. Countries such as Indonesia and India also continue to report relatively low HPV vaccination coverage, reflecting ongoing challenges related to access, affordability, awareness, vaccine acceptance, and program implementation [29,30,31].

Other examples in Asia are represented by regions with well-established programs that have obtained significantly better results than disadvantaged regions such as Myanmar or Indonesia in terms of incidence and mortality (Table 2). In contrast, marked internal variation can also be observed within the same country, as in China, where vaccination coverage has been reported at 45% in urban areas and 20% in rural areas [28,29,32,33,34,35,36]. To address these disparities, WHO recommends increasing access to free vaccination in rural areas, raising awareness, and establishing sustainable screening and treatment policies for all population groups [28,29,30,37].

Marked disparities were observed in Africa as well. Although countries like Tanzania and Kenya achieved vaccination coverage up to 80% in urban areas, many other regions have vaccination rates below 50% [37,38]. In Nigeria, prevention initiatives have been hindered by limited resources and a lack of awareness, leading to vaccination coverage below 30% [37,39,40]. A recent study concerning HPV vaccination in Kenya demonstrated that a single HPV vaccine dose remains effective over three years, potentially supporting improved vaccination coverage in resource-constrained settings [37,38].

Another significant challenge is gaining cultural acceptance for the vaccine. In Zimbabwe, research on HPV genotype distribution highlights the need to expand vaccination efforts to cover the main cancer-causing strains [37,41]. Additionally, education and awareness campaigns have played a crucial role in increasing vaccine acceptance among adolescent girls in Ethiopia, with certain regions achieving up to a 60% vaccination rate thanks to strong involvement from community leaders [37,42,43].

In the United States, socioeconomic disparities and stigma associated with HPV vaccination persist as considerable barriers for certain demographic groups, including indigenous populations and communities with restricted access to healthcare services, despite a vaccination coverage rate of 75% for girls aged 13 to 17 years [44]. Research suggests that vaccination initiatives aimed at vulnerable populations, such as indigenous women, should be culturally and logistically customized to enhance acceptance and engagement in preventive programs [45]. Canada reported an HPV vaccination rate for girls aged 14 and younger of 80% through comprehensive prevention strategies, and Mexico, with a well-organized healthcare system, reported a vaccination coverage of 60% in 2024 [46,47,48,49]. Australia remains one of the top-performing countries with HPV vaccination coverage of 80.5% [50,51,52,53,54].

### 3.3. Cervical Cancer Screening

Significant differences were also identified regarding cervical cancer screening. Western Europe uses national screening programs that include HPV testing every 5 years for women aged 30–65 years, directly improving early detection and reducing advanced cases of cervical cancer. Educational campaigns, such as those carried out in France and the Netherlands, have also contributed to improving awareness and supporting HPV vaccination uptake, despite population reluctance; all these elements underline the need for an integrated approach that combines both organized screening and campaigns dedicated to vulnerable groups [22,26,55,56,57]. Eastern Europe remains substantially behind, with disorganized screening programs, participation rates frequently below 50%, and major regional disparities [26,27].

Across Asia, both screening participation and screening modality vary considerably. In Japan, where cervical cancer screening has traditionally relied on cytology rather than primary HPV testing, participation has been reported at around 70%, whereas several countries in South and Southeast Asia continue to report substantially lower screening uptake. These differences should be interpreted with caution because HPV testing and cervical cytology are not directly comparable in terms of performance and expected impact [28,29,30,31]. In China, internal variation persists, with urban regions demonstrating significantly better screening performance than rural regions [32,33,34,35,36].

Screening in sub-Saharan Africa faces many resource challenges, including infrastructure and financial issues. A study in Ethiopia found that only 14% of women ages 30–49 have ever had a cervical cancer screening test [37,42,43]. Self-collection of samples has been seen as a promising way to overcome barriers related to stigma and limited access to healthcare facilities [37,58].

Canada, through comprehensive strategies for cervical cancer prevention, which included awareness campaigns and organized screening, has achieved a screening coverage rate of 75% for eligible women by the end of 2024 [46,47,48]. Meanwhile, Brazil has made notable progress through prevention and treatment efforts by including screening tests in its national health program, reaching about 60% coverage of eligible women, but logistical and financial challenges remain [59,60]. In Mexico, despite having a relatively well-organized healthcare system, screening coverage was reported to be below 50% in 2024 [46,47,48,49].

In Australia, between 2018 and 2021, over half of women aged 45–49 years participated in screening activities [51,52]. Since the start of organized screening in 1991, cervical cancer incidence and mortality have fallen by approximately 50%. Under the renewed National Cervical Screening Program, implemented in December 2017, primary screening is based on HPV testing, while liquid-based cytology is used mainly as a triage test for HPV-positive women [50,51,52]. In 2023, 7% of screening HPV tests were positive for oncogenic HPV, and 85% of participants with detected precancerous lesions were treated within less than half a year [50,51,52,53,54].

### 3.4. Treatment, Incidence, and Outcomes

The capacity to diagnose and treat precancerous lesions and cervical cancer differed markedly between regions. Western Europe and North America demonstrated sustained reductions in cervical cancer mortality and incidence following massive investments in the health system and cancer programs [14,15,16,17]. Economic analyses in countries like Germany and Spain show that combined vaccination and screening programs are financially sustainable over the long term, costing about 1200 euros per case prevented [22,26,27]. In contrast, in Romania, limited funding and delayed implementation continue to compromise program efficiency. According to GLOBOCAN 2022, Romania had an estimated 3368 new cervical cancer cases and 1793 deaths in 2022, corresponding to age-standardized incidence and mortality rates of 21.7 and 9.3 per 100,000 women, respectively [2,61]. These figures confirm that Romania remains among the countries with the highest cervical cancer burden in Europe [61].

Asia reflected similar contrasts. India accounts for over 120,000 new cervical cancer cases and over 77,000 deaths annually, representing nearly 17% of the global burden [30]. This situation results from over-screening and under-vaccination and insufficient access to treatment, compounded by cost barriers [30,31].

Cervical cancer continues to represent a major health burden in sub-Saharan Africa, where the age-standardized incidence and mortality rates were estimated at 33.4 and 22.6 per 100,000 women, respectively, in 2022 [2,62]. Zimbabwean data regarding HPV genotype distribution further supports the need to expand vaccination and treatment measures [41].

The same obstacles are also recorded in Latin America. A permanent confrontation with insufficient funds, ineffective campaigns, economic inequalities, and outdated medical infrastructure has led to limited access to treatment and screening in rural areas, even in regions like Mexico with a well-organized healthcare system [49].

Australia is among the best-performing countries in terms of cervical cancer management, with an incidence of 6 per 100,000 women and a mortality rate of 2 to 3 per 100,000 women [50]. All these favorable results were also obtained through the involvement of indigenous communities that contributed to improving accessibility to screening and treatment, even though participation rates among them continue to be below the national average, with incidence 2.3 times higher than in the non-indigenous population [50,51,52,53,54].

### 3.5. Low and Middle-Income Countries (LMICs)

Low-income countries, particularly Eastern Europe, South Asia, and sub-Saharan Africa, report HPV vaccination rates below 30–40%, screening participation below 30%, and significantly reduced access to treatment. These gaps are largely due to resource constraints, health system inefficiencies, socioeconomic inequalities, limited public health literacy, stigma, and inappropriate policy prioritization [26,27,28,29,30,31,37,38,39,40,41,42,43]. This disproportionate burden of cervical cancer incidence and mortality in these regions continues to reinforce global inequities and highlights the persistent gap in achieving the WHO strategy.

## 4. Discussion

### 4.1. HPV Vaccination Policies—Between Models of Excellence and Systemic Failures

One of the most important elements of the WHO strategy is HPV vaccination; however, its implementation remains very uneven across regions. The results of this analysis confirm a persistent gap between high-income and low-resource countries, a discrepancy that is largely reinforced by major differences in health system financing, organizational capacity, and public trust in preventive programs [14,15,16,17,22,23,24,25,26,27].

Countries such as Australia, Canada, the United Kingdom, and Italy demonstrate that high vaccination coverage is achievable through coordinated national programs, with reported coverage reaching 80.5% in Australia, 80% in Canada, 85% in the United Kingdom, and 71% in Italian girls, alongside increasing uptake among boys [22,23,24,25,26,27,46,47,48,49,50,51,52,53,54]. In contrast, Eastern Europe and parts of South Asia remain well below WHO targets, with vaccination rates under 30%, a pattern also observed in sub-Saharan Africa, where substantial urban–rural disparities persist (Table 3) [26,27,28,29,30,31,37,38,39,40,41,42,43].

### 4.2. Participation in Cervical Screening—Between Systematic Organization and Fragmented Opportunism

Screening participation reflects the degree of organization and integration of the health system. In countries such as Australia, the United Kingdom, and Canada, screening participation rates of over 70% have been achieved when population-based screening programs have incorporated HPV testing [26,27,28,29,30,31,46,47,48,49,50,51,52,53,54]. In contrast, fragmented and opportunistic screening persists in Eastern Europe, South Asia, and sub-Saharan Africa, where participation remains below 30–50%, contributing to delayed diagnosis and maintaining the burden of disease [26,27,28,29,30,31,32,33,34,40,41,42,43,44,45,46].

### 4.3. Treatment of Precancerous Lesions

For the effective management of cervical cancer and to reduce mortality caused by it, along with adequate screening and vaccination programs, rapid and effective treatment of precancerous lesions is necessary. Regions with integrated prevention and treatment approaches demonstrate superior outcomes, while under-resourced health systems experience delays in diagnosis and treatment initiation. Australia exemplifies effective implementation, with over 85% of detected precancerous lesions treated within six months [50,51,52,53,54]. In contrast, persistent underfunding and limited access to specialized care compromise treatment outcomes in Romania and sub-Saharan Africa [22,23,24,25,26,27,37,38,39,40,41,42,43].

### 4.4. Geographical, Economic, and Cultural Inequalities—The Invisible Barrier to Elimination

The ability of the population to have access to prevention and treatment regardless of their background and social situation is another major problem facing the global public health community. Disparities are evident between nations, regions of the same country, between communities, between urban and rural environments, and between advantaged or vulnerable social groups [14,15,16,17,26,27,37,38,39,40,41,42,43].

Even in the case of well-developed countries, with major political importance at a global level, such as the United States, where there is an advanced medical infrastructure, in terms of screening, there are still low vaccination rates and participation among indigenous communities and low-income populations due to stigma, limited access to education, language, and cultural barriers [44,45]. The same situation is also found in less developed countries such as Romania or Africa, where geographical distance, extreme poverty, and restrictive cultural norms represent significant obstacles to prevention services [22,23,24,25,26,27,37,38,39,40,41,42,43].

More broadly, the medical literature shows that severe comorbidities, delayed presentation, and limited family or social support can negatively influence access to timely diagnosis, continuity of care, and adherence to medical recommendations in vulnerable patients. Although these observations arise from different clinical contexts, they remain relevant to public health strategies that depend on early detection, follow-up, and sustained patient engagement. In this regard, they may also inform broader approaches to cervical cancer prevention, particularly in relation to high-risk groups, delayed access to care, and the need for supportive family or community involvement in underserved populations [63,64,65].

### 4.5. Romania—The Example of an Incomplete Transition

Romania illustrates the broader challenges of transitional health systems in Central and Eastern Europe. Despite EU membership, persistent underfunding, fragmented public health infrastructure, and low public trust in prevention programs have resulted in HPV vaccination coverage below 30%, participation in organized screening below 25%, and one of the highest cervical cancer mortality rates in Europe [22,23,24,25,26,27]. These findings reflect patterns observed in similar transitional systems, rather than representing an isolated national failure.

**Barriers and Challenges**

Analyzing the implementation of the WHO strategy, several common barriers were identified, especially in disadvantaged or less developed regions. These include inefficient and outdated healthcare infrastructure, limited access to HPV vaccination and testing for disadvantaged populations, low levels of health literacy in isolated communities, population reluctance influenced by misinformation, myths, or lack of trust in specialists in the field, insufficiently trained medical staff, and significant differences between urban and rural areas in terms of cervical cancer prevention.

**Opportunities and Future Perspectives**

Despite the challenges mentioned above, there are opportunities that can accelerate alignment with the WHO strategy. These include the development of international partnerships for technical, moral, and financial support, the expansion of free vaccination programs in schools, the use of self-administered HPV testing (self-sampling) for isolated or reluctant communities, the digitalization and better organization of screening programs, and the involvement of local, religious, and community leaders specific to each region, especially in risk areas.

**Limitations of the study**

Although it covers a wide range of regions and strategies, this study has several limitations. Unequal access to updated and comparable data across regions remains an important constraint. Another limitation is the methodological variability of the included sources, which comprised registry-based reports, official documents, guidelines, narrative reviews, systematic reviews, and original studies. This heterogeneity may affect the consistency of the evidence synthesis and, in some cases, limits direct comparison between settings. In addition, key indicators such as vaccination coverage, screening participation, and treatment outcomes were not uniformly defined or reported across countries. Programs implemented close to December 2024 could not be adequately evaluated because outcome data were not yet available. Data extraction was performed by a single reviewer, which may introduce selection bias, although all extracted information was subsequently checked for accuracy by a senior team member to ensure the consistency and reliability of the final dataset.

## 5. Conclusions

Based on the data collected during this study, it is confirmed that eliminating cervical cancer is an attainable goal. The WHO strategy, rooted in three core elements—screening, vaccination, and treatment—offers a standardized yet flexible framework. This promotes a public health strategy centered on prevention, equity, and efficiency, regardless of socioeconomic status.

The findings in this review highlight a clear connection between health system development and successful implementation of the WHO strategy. Countries such as Australia and Canada have achieved more favorable results through sustained investment in vaccination programs, organized screening, diagnostic infrastructure, and public health campaigns. In contrast, many settings in sub-Saharan Africa and Latin America continue to face major barriers related to socioeconomic inequalities, limited resources, and unequal access to prevention and care.

Another negative example is Romania, which, although a member of the European Union that can benefit from all the key elements necessary to eliminate cervical cancer, continues to report concerning figures regarding its incidence and mortality rates. Romania remains far from the WHO goals and risks falling behind in prevention, with a vaccination rate below 30% and a screening participation rate under 25%.

Therefore, eradicating cervical cancer is no longer just a hope but a proven reality in some countries. However, to ensure this progress is not limited locally, a clear delineation of responsibilities is essential—one that involves political leaders, healthcare professionals, and citizens alike—to turn the fight against cervical cancer from a desperate struggle into a successful national and global effort.

## Figures and Tables

**Table 1:** Characteristics of the publications included in the review.

Authors/Year	Country/Region	Source Type	Study Population/Dataset	Main Contribution
International Agency for Research on Cancer	Global	Fact sheet	Global estimates	Global cervical cancer burden
Bray, F.; et al., 2024	Global (65 countries)	Registry-based dataset	460 cancer registries	Global cervical cancer incidence burden
Peto, J.; et al., 2004	United Kingdom	Epidemiological study	National population data	Impact of organized screening on cervical cancer burden
Ronco, G.; et al., 2014	Europe	Randomized trials	Four European trials	Effectiveness of HPV-based screening
WHO Regional Office for Europe, 2021	Europe	WHO recommendation	Women eligible for screening	WHO recommendations for HPV-based screening
Sankaranarayanan, R.; et al., 2009	India	Randomized trial	Rural women in India	HPV screening effectiveness in a rural setting
Debbie, S.; et al., 2016	United States	Guideline update	Females and males aged 9–26 years	HPV vaccination effectiveness and endorsement
World Health Organization, 2020	Global	Strategy document	Global target population	WHO 90-70-90 cervical cancer elimination targets
Bruni, L.; et al., 2023	Global	Epidemiological report	Global female population ≥ 15 years	Global cervical cancer burden and HPV-related disease
Julie, T.; et al., 2021	Global	Public health analysis	Global population; girls aged 9–14 years	WHO strategy implementation and global inequities
World Health Organization, 2014	Global	Guidance document	Women eligible for prevention and care	Comprehensive cervical cancer control guidance
World Health Organization, 2022	Europe	Official report	WHO European Region	European roadmap of cervical cancer elimination
Supitcha, K.; et al., 2021	Thailand	Narrative review	Adolescents and adults aged 9–45 years	HPV vaccine efficacy and effectiveness
Abate, W.T.; et al., 2024	Unspecified	Narrative review	Women at risk of cervical cancer	Cervical cancer prevention and challenges
Jomah, A.; et al., 2024	Saudi Arabia	Systematic review	19 studies; women aged 18–75 years	Impact and effectiveness of HPV vaccination
Ying, L.; et al., 2024	China	Narrative review	Women at risk of cervical cancer	HPV pathophysiology, screening, and vaccination
Castle, P.E.; et al., 2024	United States	Narrative review	LMIC and disadvantaged HIC population	Global implementation challenges and opportunities
Alvine, M.A.; et al., 2022	Global	Systematic review	Adolescents aged 9–18 years and high-risk adults	HPV vaccine delivery strategies and costs
Xia, C.; et al., 2024	China	Narrative review	Over 100 articles	HPV vaccine strategies and elimination progress
Gaia, G.; et al., 2022	Global	Narrative review	Global populations at risk; girls aged 9–14 years	HPV vaccination and cervical cancer elimination
Li, S.; et al., 2024	Europe	Systematic review	Women eligible for screening; 17 studies	Cost-effectiveness of screening interventions
Sunyoung, C.; et al., 2023	United Kingdom	Narrative review	Women aged 25–64 years	UK cervical cancer epidemiology and screening
Giorgio, B.; et al., 2023	Italy	Narrative review	Italian girls and boys aged 11–12 years	HPV vaccination coverage and barriers in Italy
Giovanni, G.; et al., 2021	Italy	Narrative review	Italian target groups	HPV vaccination coverage and strategies in Italy
Klejda, H.; et al., 2024	Europe	Systematic review	European screening populations	Cost-effectiveness of cervical cancer screening in Europe
Ministry of Health, 2023	Romania	Policy document	National Cancer Control Framework	Romanian national cancer control strategy
Rei, H.; et al., 2021	Western Pacific Region	Narrative review	17 middle-income countries	HPV vaccination implementation
Bugra, T.G.; et al., 2024	Middle East	Systematic review	159 studies	HPV vaccine awareness and acceptance
Sasidharanpillai, S.; et al., 2024	South Asia	Systematic review	Women eligible for screening: 23 studies	Cervical cancer screening strategies in South Asia
Vinit, N.D.; et al., 2024	India	Narrative review	Women and adolescent girls in India	HPV burden, prevention, and vaccination in India
Asami, Y.; et al., 2021	Japan	Narrative review	Girls aged 12–16 years and women in Japan	Impact of HPV vaccine recommendation suspension
Wenchuan, S.; et al., 2021	China	Systematic review	Women and girls eligible for HPV vaccination	Cost-effectiveness of HPV vaccination in China
Chao, Z.; et al., 2024	China	Narrative review	The general female population in China	Barriers and opportunities for HPV vaccination in China
Lihui, W.; et al., 2021	China	Narrative review	Chinese female population aged 9–45 years	Immunogenicity, efficacy, and safety of HPV vaccines
Ryan, C.V.; et al., 2022	Philippines	Narrative review	Population of the Philippines	HPV burden, screening, treatment, and vaccination
Laia, B.; et al., 2023	Global	Systematic review/meta-analysis	Men from the general population aged ≥15 years	Global prevalence of genital HPV infection in men
Ruanne, V.B.; et al., 2023	Kenya	Randomized trial	2275 participants	Durability of single-dose HPV vaccination
Alex, M.; et al., 2022	Tanzania	Implementation study	14-year-old girls in Tanzania	National HPV vaccine introduction in Tanzania
Ishak, K.L.; et al., 2021	Nigeria	Narrative review	Nigeria population	WHO strategy implementation challenges in Nigeria
Takudzwa, M.; et al., 2024	Zimbabwe	Narrative review	21 studies and national HPV vaccination data	HPV genotype distribution in Zimbabwe
Amare, Z.; et al., 2023	Ethiopia	Systematic review/meta-analysis	Adolescent girls; 7 studies	HPV vaccine acceptance in Ethiopia
Dagne, A.; et al., 2023	Ethiopia	Systematic review/meta-analysis	Adolescent girls; 13 studies	HPV vaccine knowledge, attitude, and uptake
Hanna, A.T.; et al., 2021	Sub-Saharan Africa	Systematic review/meta-analysis	8200 participants aged 25–65 years	Effectiveness of HPV self-sampling
Caryn, E.P.; et al., 2021	United States	Scoping review	Women and the general US population	Stigma and cervical cancer prevention
Crystal, G.M.; et al., 2022	United States	Narrative review	Native American populations	HPV disparities in Native American women
Samara, P., 2024	Canada	Narrative review	General population of Canada	Progress and challenges in Canada
Anna, R.G.; et al., 2023	United States	Narrative review	General population of the United States	US progress toward cervical cancer elimination
Angélica, N.R.; et al., 2022	Latin America	Narrative review	Girls/teens eligible for vaccination	HPV vaccination coverage and barriers in Latin America
Eder, A.B.; et al., 2022	Mexico	Narrative review	Women with cervical cancer in Mexico	Prevention, screening, and treatment access in Mexico
Department of Health and Aged Care, 2023	Australia	Strategy document	National cervical cancer control framework	National strategy for cervical cancer elimination
World Health Organization, 2021	Australia	Country profile	National estimates for Australia	Australia cervical cancer profile and burden indicators
Australian Institute of Health and Welfare, 2024	Australia	Monitoring report	National screening program data	National cervical screening program outcomes in Australia
Lisa, J.W.; et al., 2021	Oceania and North America	Narrative review	Indigenous and overall women in four countries	Progress toward elimination in indigenous women
Cristyn, D.; et al., 2021	Australia	Qualitative study	Parents of adolescents eligible for HPV vaccination	Parental attitudes toward HPV vaccination in Australia
Wei, W.; et al., 2022	Global	Systematic review	Women eligible for screening in 11 countries	Comparison of cervical screening guidelines and programs
Géraldine, E.B.; et al., 2021	France	Systematic review	25 studies	General practitioners’ views on HPV vaccination
Maver, P.J.; et al., 2020	Europe	Narrative review	Women eligible for screening in Europe	Implementation of HPV-based screening in Europe
Flávia, M.C.; et al., 2022	Brazil	Narrative review	Girls aged 9–14 years and women aged 25–64 years	Screening, treatment, and vaccination in Brazil
María, L.S.; et al., 2023	South America	Systematic review	National recommendations from 13 South American areas	HPV vaccination and screening recommendations in South America
Bray, F.; et al., 2024	Global	Epidemiological report	36 cancers in 185 countries	Global cancer incidence and mortality estimates
International Agency for Research on Cancer, 2024	Romania	Fact sheet	National estimates for Romania	Romania cervical cancer incidence and mortality estimates
International Agency for Research on Cancer, 2024	Sub-Saharan Africa	Fact sheet	Regional estimates for Sub-Saharan Africa	Sub-Saharan Africa cervical cancer burden estimates

**Table 2:** Incidence and mortality rates per 100,000 women. Source: IARC/WHO Global Cancer Observatory (GLOBOCAN 2022), ASR (World) per 100,000.

	Japan	South Korea	Myanmar	Indonesia
Incidence	9.2	8.6	21.4	23.3
Mortality	2.6	2.0	13.4	13.2

**Table 3:** HPV vaccination rates by region. The table shows the low level of vaccination coverage in Eastern Europe, compared to Western areas.

Region	HPV Vaccination Rate (%)
Western Europe	85
Eastern Europe	30
North America	75
South America	60
Asia	37
Africa	50
Australia	80

## Data Availability

The data supporting the findings of this study are available from the sources cited in the reference list.

## Supplementary Materials

The following supporting information can be downloaded at: https://www.mdpi.com/article/10.3390/diagnostics16081224/s1. Table S1: PRISMA 2020 Checklist.
