# Correction: Luca et al. Global and Regional Diagnostic Results of Progress Toward Cervical Cancer Elimination, According to the WHO Strategy: A Systematic Literature Review with Narrative Synthesis. *Diagnostics* 2026, *16*, 1224

**DOI:** 10.3390/diagnostics16121764

**Published:** 2026-06-08

**Authors:** Dan Cristian Luca, Ciprian Cirimbei, Sinziana Octavia Ionescu, Vlad Rotaru, Dan Nicolae Straja, Mihnea Alecu, Elena Chitoran, Daniela Cristina Stefan, Laurentiu Simion

**Affiliations:** 1 Medicine School, “Carol Davila” University of Medicine and Pharmacy, 050474 Bucharest, Romania; dan-cristian.luca@drd.umfcd.ro (D.C.L.); ciprian.cirimbei@umfcd.ro (C.C.); sinziana.ionescu@umfcd.ro (S.O.I.); vlad.rotaru@drd.umfcd.ro (V.R.); dannicolaestraja@gmail.com (D.N.S.); mihnea.alecu@umfcd.ro (M.A.); elena.chitoran@drd.umfcd.ro (E.C.); laurentiu.simion@umfcd.ro (L.S.); 2 General Surgery and Surgical Oncology Department I, Bucharest Institute of Oncology “Prof. Dr. Al. Trestioreanu”, 022328 Bucharest, Romania

## 1. Methodological Clarification

Following the post-publication review process, the authors wish to provide an additional clarification regarding the methodological approach used in this study.

The original publication [1] was conducted using a structured literature search strategy across multiple international databases, combined with predefined inclusion criteria. Through this process, 47 studies were identified through the PRISMA-based selection process and included based on their direct relevance to the implementation of the WHO cervical cancer elimination strategy. These studies represent the core evidence base used for the primary analysis.

In addition to these studies, Table 1 presents a broader set of publications (total *n* = 62), comprising not only the studies selected through the PRISMA-based process, but also supplementary sources such as narrative reviews, systematic reviews, institutional reports, guidelines, and epidemiological datasets. As a result, the article uses a heterogeneous mix of primary and secondary sources.

These supplementary sources were incorporated to support contextual interpretation, enable comparison between regions, and provide a more comprehensive understanding of the available evidence. Review articles and other secondary sources were not used as primary evidence for assessing progress toward the WHO 90-70-90 targets. Rather, their role was complementary, contributing to the interpretation and discussion of findings.

## 2. Correction of Article Type and Title

Given this approach, the study should be interpreted as a systematic literature review using narrative synthesis, in which the PRISMA-selected studies formed the core evidence base and supplementary sources were used for contextual and interpretative support. Accordingly, the article should not be read as a conventional systematic review based mainly on primary studies. The article type is changed from “Systematic Review” to “Review”, and the title is changed from “Global and Regional Diagnostic Results of Progress Toward Cervical Cancer Elimination, According to the WHO Strategy: A Systematic Review” to “Global and Regional Diagnostic Results of Progress Toward Cervical Cancer Elimination, According to the WHO Strategy: A Systematic Literature Review with Narrative Synthesis”.

This clarification is intended to improve transparency regarding the methodological framework and should be considered when interpreting the Methods section, Table 1, and the overall structure of the study. Because of the heterogeneous nature of the evidence base and the inclusion of secondary and contextual sources, the conclusions should be interpreted with appropriate caution.

This clarification does not alter the data presented or the overall conclusions of the article. The authors state that the scientific conclusions are unaffected. This correction was approved by the Academic Editor. The original publication has also been updated.
